# Understanding Infantile Atopic Dermatitis: A Review of Environmental, Familial, Genetic and Microbial Influences

**DOI:** 10.1007/s11882-026-01272-7

**Published:** 2026-05-20

**Authors:** Sasha McKay, Xiu-Min Li

**Affiliations:** 1 School of Medicine, New York Medical College, Valhalla, NY USA; 2 Department of Pathology, Microbiology & Immunology, School of Medicine, New York Medical College, Valhalla, NY USA; 3 Department of Otolaryngology (ENT), New York Medical College, Valhalla, NY USA; 4 Department of Dermatology, New York Medical College, Valhalla, NY USA

**Keywords:** Eczema, Environmental factors, Gut Microbiome, Infantile Atopic Dermatitis, Skin Microbiome

## Abstract

**Purpose of Review:**

This review aims to clarify the early-life risk and protective factors associated with Infantile Atopic Dermatitis (IAD)—an inflammatory skin condition that typically develops between birth and two years of age. The goal was to examine recent findings on maternal, environmental, and microbial influences on IAD.

**Recent Findings:**

Prenatal and postpartum maternal probiotic use may reduce IAD risk, though no significant alterations in infants’ gut microbiota were found. Infants with IAD exhibit higher *Clostridia* levels, while *Verrucomicrobi*a are more abundant in non-IAD cases. Breastmilk from the mother of affected infants contains higher arachidonic acid and lower eicosapentaenoic acid, whereas formula-feeding may lower IAD risk. Seasonal influences such as reduced sunlight or humidity are associated with higher susceptibility. Elevated skin biomarkers, including TARC/CCL17 and IL-8, have been observed in infants who later develop IAD. Early antibiotic exposure, particularly during the first trimester, also increases risk.

**Summary:**

IAD is multifactorial, involving genetics, environment, and skin barrier dysfunction. Understanding the interplay between the microbiome, maternal influences, and environmental exposures may guide future preventive approaches. Further research into non-pharmacologic and microbiome-targeted interventions is warranted to delay or prevent IAD onset.

## Introduction

Atopic Dermatitis (AD) is an inflammatory skin disorder that affects roughly 30% of the pediatric population worldwide and can persist into adulthood, while infantile AD (IAD) typically manifests between birth and 2 years of age [1–3]. It typically presents as pruritic, exudative, erythematous papules and vesicles on the face or scalp and may spread to the trunk and extensor surfaces of the extremities [2]. Children affected by infantile AD often experience reduced quality of life due to sleep disturbances caused by intense pruritus [2]. Some of the pathogenesis of AD is thought to involve interactions between genetics, skin barrier dysfunction, alterations in the skin microbiome, and environmental exposures [4]. The risk of developing AD is higher in children with a parental history of allergic disease compared to those with no parental history, and the risk is present if a mother, rather than the father, has a history of AD or allergies [5, 6]. Other familial factors include smoking during pregnancy, having a sibling with atopy, and low food diversity [7, 8].

Some studies suggest that dysbiosis in the gut and skin flora may be associated with AD [9–11]. Numerous studies have suggested that there are differences in the gut and skin microbiomes of infants with AD and healthy infants [7, 12, 13]. Furthermore, peer-reviewed findings have shown that even the composition of fats and cytokines in the breastmilk of mothers with AD infants varies from the breastmilk of mothers with healthy infants [9, 14]. Recent investigations have looked at whether the season, location, and month of birth also play a role in the pathogenesis of AD and have found that there may be an association depending on when and where the birth occurs [5, 15]. Other studies have investigated whether there are differences in the skin of infants with AD and healthy infants that could help serve as biomarkers [16, 17]. Previous research has also suggested that exposure to antibiotics in utero and early life has been associated with an increased risk of developing AD [4, 18, 19].

Previous studies on AD typically did not involve examining the risk factors of infantile AD. Much of the recent research on AD focuses on personalized treatment that involves the microbiota and how this influences the gut-skin microbiota, as well as the potential benefits of probiotics, which were not explicitly discussed in regards to infantile AD [20, 21]. The infantile skin plays an important role in both protecting against pathogens and priming the immune system; there are also some slight differences in the skin microbiome between adults and infants [22].

AD plays a vital role in the development of the atopic march and in the progression of other atopic diseases such as IgE-mediated food allergies, allergic rhinitis, and asthma [23]. Typically, AD develops first, followed by the onset of these other atopic conditions, thereby increasing the risk of their development. In fact, the incidence of food allergy onset is six times higher in children with AD compared to those without, and IgE antibodies can be detected in infants as early as 1 month of age [23]. Sensitization often occurs before oral intake, and the rate of cutaneous sensitization increases with the severity of AD. Additionally, the prevalence of asthma also rises with increasing AD severity [23]. Understanding these environmental factors, such as gut flora, breast-milk composition, skin biomarkers, and antibiotics, could alleviate the stress that infantile AD may cause and could help guide new early interventions to prevent the progression into a more severe disease.

## Gut-Microbiome

**Fig. 1:**
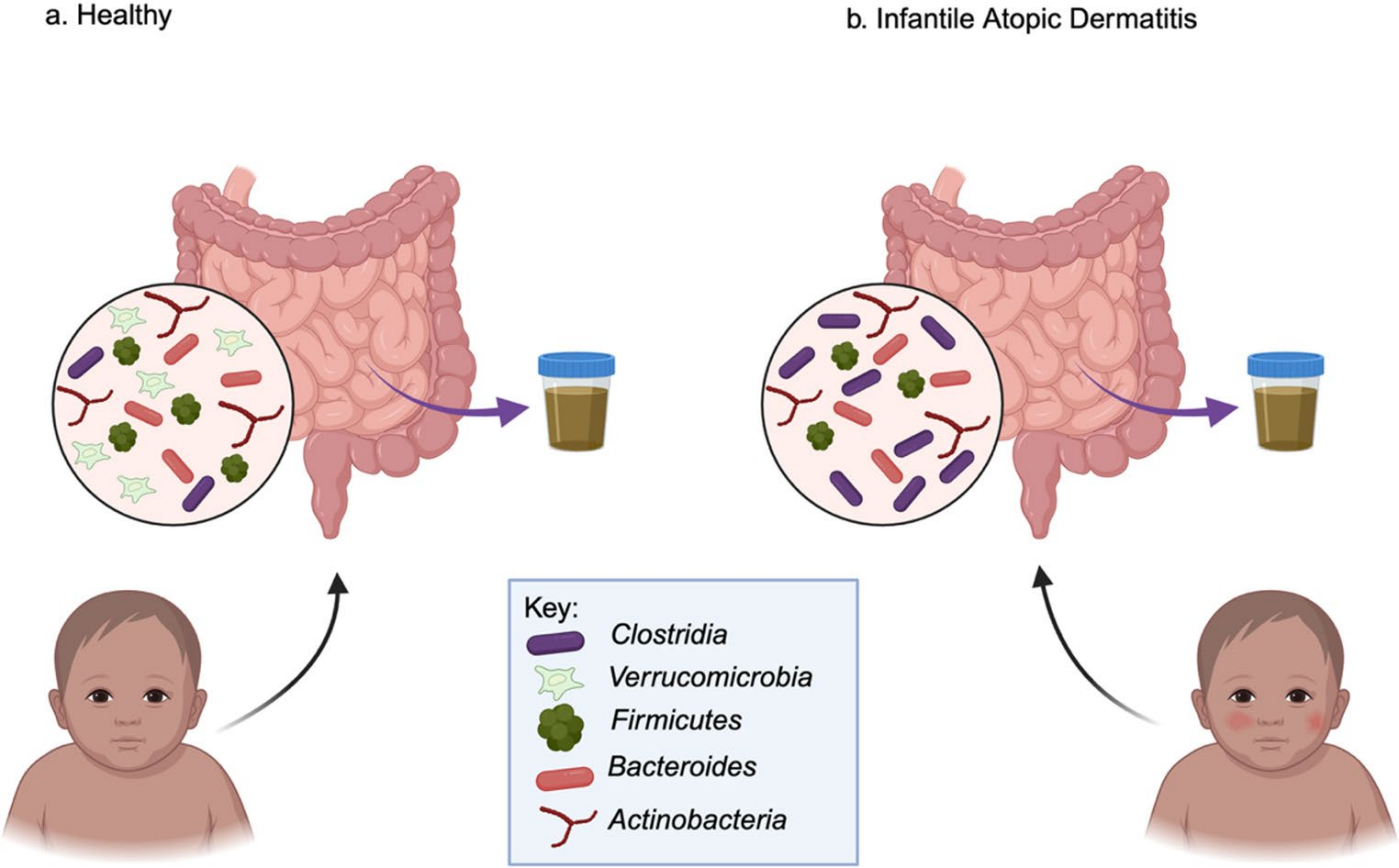
Summary of Gut Flora Composition in Healthy Individuals and Infantile AD. (**a**) The composition of bacteria in infants without AD includes *Clostridia*, *Verrucomicrobia*, *Firmicutes*, *Bacteroides*, and *Actinobacteria*. (**b**) The composition of bacteria in infants with AD is like healthy individuals; instead, they lack *Verrucomicrobia* and have a higher abundance of Clostridia. (Created with BioRender)

The gut microbiome may factor into the pathogenesis of infantile AD. A few studies explored the composition of the maternal and infant microbiome with its association with AD. It was demonstrated that when breastfeeding mothers in mother-infant pairs were given probiotics, 2 months before birth and 2 months postpartum, that contain *Lacticaseibacillus rhamnosus LPR* and *Bifidobacterium longum BL999*, or *Lacticaseibacillus paracasei ST11* and *Bifidobacterium longum BL999*, versus a placebo, infants in the placebo group developed AD at higher rates when compared to the other groups (*p* = 0.0008) [12]. It is also worth noting that although there were no significant differences in gut microbiome changes between 1 month and 6 months of age in all of the groups, there was still a higher level of *Clostridia* in the *infantile* AD (IAD) group. This suggests that although probiotics may help reduce the risk of IAD, they did not significantly alter the overall microbial diversity of infants, indicating that other mechanisms may be involved [12]. Another similar study examined the maternal gut-microbiome of mother-infant pairs and observed that mothers of infants with AD had significantly higher abundance of Proteobacteria than the mothers within the healthy control group (0.37% and 0.09% respectively) [13]. Additionally, *Verrucomicrobia*, specifically *Akkermansia muciniphila*, was found only in the maternal healthy control group (1.38%, *p* = 0.0004) and in the infant healthy control group [13]. Furthermore, *Firmicutes*, *Bacteroides*, and *Actinobacteria* were present in all the groups [13]. It is also noteworthy to mention that, at 2 years old, the risk of developing AD was higher in colic infants (40%) than the healthy infants (20%) [24]. Together, these findings suggest that the differences in intestinal flora of healthy infants and infants with AD could contribute to an underlying inflammatory mechanism that could lead to IAD and that treatment with probiotics could reduce the risk of developing IAD. A visual summary of the composition of the gut microbiome of healthy infants and IAD infants can be seen in Fig. 1.

### Maternal and Infant Diet

Maternal diet, breastmilk-derived lipids, immunomodulatory cytokines, and microbes may influence AD pathogenesis. Current AAAAI/ACAAI guidelines strongly recommend against elimination diets in infants because of the potential harm associated with prolonged avoidance of food allergens and the minimal improvements in AD these diets provide [25]. A meta-analysis by the EAACI task force offers several infant dietary strategies that may help improve AD outcomes, including using probiotics for more than 12 weeks, initiating probiotics after 36 months of age, avoiding probiotics that contain *Lacticaseibacillus rhamnosus*, and adding prebiotics to the probiotics [26]. The EAACI also recommends Vitamin D, which shows dose-independent benefits in AD, as well as MUFAs and omega-3 and omega-6 fatty acids, given the deficiency of these nutrients in AD pathogenesis. Greater dietary diversity within the first year of life has been associated with reduced food allergen sensitization and may contribute to a lower risk of developing allergic disease [27].

Several studies have identified differences between the breast milk of infants with AD and those without AD. A recent study investigated the relationship between breast milk-derived metabolites and gut dysbiosis in infants with and without AD and revealed that the concentration of arachidonic acid (AA) was significantly higher in the AD group compared to the healthy controls (AUC = 0.662, *p* = 0.038) [9]. In contrast, levels of eicosapentaenoic acid (EPA) (AUC = 0.501, *p* = 0.009), 18-hydroxy-eicosapentaenoic acid (18-HEPE) (AUC = 0.501, *p* = 0.042), and DHA (AUC = 0.559, *p* < 0.001) were significantly lower in the AD group [9]. Further analysis revealed that even low concentrations of AA significantly altered the gut microbiome at 1 month of age (*p* < 0.001), promoting dysbiosis-associated changes, while EPA exerted more limited and distinct effects on the microbiome at 6 months and did not induce comparable dysbiotic shifts (*p* < 0.001). To add to this, increased sterols present in breastmilk, such as Cholestanol, Lanosterol, Lathosterol, and Stigmasterol, were associated with a reduced odds ratio (OR) of AD in infants [28]. Additionally, analysis of human breast milk samples revealed non-significant reductions in TIE2 and CD27 levels in mothers of infants with AD [14]. However, both alpha and beta diversity of the breast milk microbiota differed significantly between the AD and non-AD groups, suggesting a potential role for milk microbiota in early immune modulation [14]. It is also worth mentioning that decreased breastfeeding and increased formula feeding were associated with reduced AD morbidity [9]. Additionally, a meta-analysis assessing formula-based interventions for AD revealed that at a six-month follow up, thickened amino acid-based formula (TAAF) led to an 11.85 point reduction the SCORAD scores (95% CI: −15.68 to −7.94; low certainty), while standard amino acid formula (AAF) resulted in a 5.50-point reduction (95% CI: −9.20 to −1.82; low certainty), both compared to extensively hydrolyzed formula (EHF) [29].

Maternal diet influences the development of AD in infants. Currently, mothers are strongly encouraged to consume a diet that includes fish, fruit, and vegetables [30]. Venter et al. (2017) found that maternal consumption of fish, leafy vegetables, and fruits such as apples during pregnancy has a protective effect against the development of AD, whereas vegetables such as celery increase the risk of AD. They also reported conflicting evidence regarding the association between citrus fruits and AD. In addition, they suggested that maternal intake of n-3PUFA lowers the risk of developing AD in infants [30]. A higher healthy diet diversity during pregnancy reduces the risk of AD in offspring, whereas unhealthy dietary patterns may increase risk [27]. Furthermore, a maternal diet during pregnancy characterized by high poultry consumption was associated with a higher incidence of AD compared with other protein sources, such as red meat and plant-based proteins [31]. Collectively, these findings suggest that the maternal diet during pregnancy is important in the development of AD.

## Meteorological Variables

Recent studies suggest the season and climate in which you are born, as well as the climate of where you live, may influence the risk of developing infantile AD. Multiple studies reported that fall births had the highest increased risk of AD, with summer and winter also having increased risk [5, 32–34]. Although, Tsuchida et al. (2023) reported this same increased risk of AD for these seasons, they also found that winter births did not have a significant association. Additionally, cold weather has been reported as a trigger for AD and for increased pruritus and skin irritability [34]. Cold weather reduces skin hydration and augments the skin’s irritant response by increasing inflammatory factors such as IL-1β, thymic stromal protein, and TNF-α, leading to a deficiency in epidermal proteins and a dysfunctional skin barrier [34].

Another study suggested that there was a lower incidence of (IE) during the summer and a higher incidence during the spring, and that infants born in the spring had a lower frequency of AD [15]. When broken down by month, infants born between October and December had the highest incidence of AD during the 3 years of follow-up, and infants born in April to June had the lowest incidence of AD (*p* < 0.0001) [6]. Evidence from another study suggested that infants born in July had the highest risk of AD at one month (aOR 1.25; 95% CI: 1.16–1.35), infants born in November had the highest risk of AD at six months (aOR 2.51, 95% CI: 2.32–2.73), and infants born in October had the highest risk of AD at 1 year (aOR 1.17, 95% CI: 1.07–1.27), compared to infants born in May [5].

Another aspect worth considering is the meteorological variables of the regions in which the infants were born or live. For example, when comparing high and low sunshine duration, there was no significant difference in the incidence of AD (*p* = 0.76) [6]. These findings stand in contrast to those of another study that suggested that higher sunshine duration was associated with reduced risk of AD at both 3 months and 6 months of age (HR 0.98 and 0.95, respectively) and that reduced sunshine duration was associated with increased hazard ratios (HRs) [35]. Conversely, the measures related to sunshine duration, sunshine percentage, and solar radiation showed no consistent or statistically significant associations across seasons [35]. Although when the sunshine duration and humidity were then further combined, infants who were in regions with both short duration of sunshine and low humidity had the highest cumulative incidence over three years when compared to infants who had a long duration with high humidity (*p* = 0.023) [6].

Expanding on these variables, low temperature, low maximum, and minimum temperatures were each associated with increased risk of developing AD [35]. Across all seasons, higher average, maximum, and minimum temperatures consistently showed significantly reduced hazard ratios (HRs ranging from 0.64 to 0.87), indicating a protective effect [35]. Consistent with this, another study suggested that there is a negative association between IE and air temperature (adjusted incidence rate ratio = 0.75; 95% confidence interval 0.59–0.94) [15]. A related study also reported that greater temperature variability was consistently associated with a slightly increased risk of AD across all seasons (HRs = 1.01–1.06) [35]. These associations are consistent with the proposed mechanism underlying seasonal worsening of AD.

AD tends to worsen during the winter because transient receptor potential (TRP) channels on various epidermal cells, which allow Ca^2+^ influx and help maintain the skin barrier, are affected by cold. Specifically, TRPV1, expressed on nociceptive receptors, can be activated by low temperatures [34]. There are also several proposed mechanisms for the exacerbation of AD in warm weather. Warmer temperatures can trigger pruritus through TRP channels, which promote the release of pruritogens such as TSLP, prostaglandin E2, and nerve growth factor. This can create a cycle of itching and scratching that further worsens AD. Additionally, heat can increase inflammation through proinflammatory cytokines like IL-1β and TSLP, which promote the Th2 response characteristic of AD; this can lead to erythema, edema, and flares. Heat can also compromise the skin barrier through increased transepidermal water loss, resulting in cracked, more permeable, and more sensitive skin that is easily irritated and prone to flares.

Another key variable to consider is humidity. One study suggested that high humidity was associated with a higher incidence of AD (*p* = 0.010) when compared to low humidity [6]. These findings are not entirely consistent. For instance, in some seasonal analyses, humidity and wind velocity also showed a protective association in most seasons, particularly from January to March and July to September [35]. When assessed independently, low humidity was also linked to increased risk of AD (HR 1.19) [35].

Pressure is an additional factor to consider. Atmospheric pressure was positively associated with IE (1.31; 95% CI 1.04–1.66) [15]. Conversely, other studies demonstrate that atmospheric pressure was associated with reduced risk (HR 0.85) [35]. In regard to vapor pressure, when compared to higher vapor pressure, lower vapor pressure was associated with increased risk of AD at 6 months (HR 1.26) [35]. Furthermore, higher vapor pressure was associated with decreased risk across all birth seasons (HR 0.65–0.83) [35]. Although focusing on a slightly different aspect, the combined effects showed that infants exposed to low temperature and low vapor pressure had the highest risk (HR 1.26), while high temperature and high vapor pressure served as the reference group with the lowest risk [35]. Notably, low temperature with high vapor pressure was associated with a reduced risk (HR 0.74), suggesting a potential interaction between thermal and moisture-related conditions in modulating AD risk [35].

In terms of precipitation, there was a negative association between IE and precipitation (0.74; 95% CI 0.58–0.93) [15]. This finding aligns with another study that suggests higher precipitation was associated with lower risk in three out of four seasons, especially in autumn (HR 0.89) [35]. These studies suggest that there may be some association with location, month, and season of birth that could factor into the pathogenesis of AD.

## Biomarkers and Skin Microbiome

A growing body of literature suggests that distinct skin biomarkers may be present before the clinical onset of AD. Several studies have identified molecular differences in clinically normal infant skin that precede disease development. For example, TARC/CCL17 and [DS]-(d17:0) levels were significantly higher in infants who developed AD within the first year, compared with those who did not (*p*-values: 0.01 and 0.02, respectively) [16]. Consistent with these findings, a separate study also showed elevated CCL17, along with cytokines CXCL10, IL-8, IL-17a, CCL2, TNF-β, IL-18, IL-22, and VEGF, in the skin of infants who later developed AD (*p* = 0.001–0.05) [11]. In addition, the lipids P-(t18:0) and CER[S]-(d20:1) were significantly lower in infants who developed AD within the 1 st year (*p* = 1 × 10^− 6^ and 0.05, respectively) [16]. Another study found that cytokines such as TSLP and IL-13 are predictive of future AD onset [36]. IL-13 and IL-4 inhibit fatty acid elongation, promoting a shift toward the shorter-chain ceramides observed at 6 weeks [37]. In preclinical AD infant skin at 6 weeks, abnormalities in the cornified lipid envelope were detected before clinical onset [37]. Early-skin changes in infants also include reduced levels of protein-bound ceramides before AD develops [36–38]. Additionally, some studies report that filaggrin breakdown products are not predictive of AD, whereas others suggest they may be. Beyond cytokines and lipids, overall alpha microbial diversity of the skin is reduced before the clinical onset of AD [39].

There is also growing evidence that cytokines profiles differ between AD and healthy skin after the clinical onset of the disease. Infants with AD had SCORAD scores that were positively correlated with IL-21 in lesional skin and IL-26 in nonlesional skin (*p* = 0.016 and 0.044, respectively), while negative correlations were observed for IL-34, PPL, LCN2, and CCL22 (*p* = 0.016–0.041) in lesional skin, and IL-1RL1 and IL-37 in nonlesional skin (*p* = 0.049) [17]. Interestingly, a skin model has shown that stable allergens, such as Pru p 3, which is the primary allergen found in peaches, may penetrate a compromised skin barrier, initiating sensitization and altering immune response [40]. These differences in the skin of healthy infants and infants diagnosed with AD suggest that some of these cytokines play a protective role in the development of AD, while others may be a predisposing factor.

Additionally, some studies suggest there may be differences in the skin flora in healthy infants and infants with AD. The commensal skin microbiome includes *Acinetobacter*,* Corynebacterium*,* Cutibacterium*, *Gemella*, *Staphylococcus*, and *Streptococcus* [39, 41]. It’s been established that *Staphylococcus aureus* plays a significant role in skin dysbiosis, resulting in an overall reduction in microbial diversity and the development of AD [39, 41–43]. The overgrowth of *S. aureus* in preclinical AD may be driven by a compromised skin barrier and decreased levels of filaggrin in infant skin [44]. However, other studies have noted that *S. aureus*, along with other *Staphylococcus* species, did not dominate the skin microbiome in infants with AD [11, 45]. This contrasts with findings that indicate children who later develop AD have increased levels of *Staphylococcus epidermidis* and *Staphylococcus hominis* and decreased levels of *Streptococcus vestibularis* and *Moraxella catarrhalis* on their skin [11]. Furthermore, these differences were still present at the time of diagnosis of AD. Additionally, in children under 3 years who later develop AD, the skin microbiome was characterized by a decrease in *Prevotella* abundance and an increase in *Fusobacteria*, *Candida*, and *Saccharomyces* [10].

Given that some of these microbes are present in the skin before a clinical diagnosis of AD, this provides valuable information for several interventions that could help prevent the development of AD. Studies are now investigating ways to manipulate the skin microbiome, as there is an association between the skin microbiome and inflammatory and repair pathways. Myles et al. (2020) treated AD skin with *Roseomonas mucosa* and observed decreased SCORAD scores and clinical improvement at several sites, including the antecubital and popliteal fossae [46]. Ongoing clinical trials are investigating the safety and efficacy of *S. hominis* in *S. aureus* colonized AD skin in patients 12 years and older [47].

## Antibiotics

Growing evidence suggests that antibiotics may influence the development of AD. This can be seen following the increased risk of developing AD after *in utero* antibiotic exposure [HR 1.38, 95% CI (1.36–1.39)] [4]. Among antibiotic classes, *in utero* exposure to penicillin had the highest increased risk at 1.43 (95% CI: 1.41–1.44), compared to Sulfa antibiotics [HR 1.24, 95% CI (1.03–1.22)], cephalosporins [HR 1.35, 95% CI (1.32–1.37)], and macrolides [HR 1.29, 95% CI (1.21–1.37)] [4]. Among mothers who received Group B Streptococcal-Intrapartum Antibiotic Prophylaxis (GBS-IAP), which included penicillin G, ampicillin, or cefazolin, and delivered vaginally, the incidence of AD in infants was 45%, compared to 11.1% among mothers who did not receive GBS-IAP was 11.1% [18]. Univariate logistic regression analysis showed that GBS-IAP in the setting of vaginal delivery was associated with an odds ratio of 6.719 of AD (95% CI: 4.730–9.544; *p* < 0.001) compared to vaginal delivery without [18]. Similar results were present in another study where antibiotic use during pregnancy was associated with an increased risk of developing childhood AD of 1.12 (95% CI: 1.11–1.13) [19]. Moreover, when stratified by timing of exposure, the highest risk was observed with antibiotic administration during the 1 st trimester [HR 1.13, 95% CI (1.12–1.14)], followed by 2nd trimester [HR 1.07, 95% CI (1.04–1.09)] and 3rd trimester [HR 1.06, 95% CI (1.04–1.09)]. Further stratification by cumulative dose revealed that children whose mothers received antibiotics more than three times during the pregnancy had the greatest risk [HR 1.20, 95% CI (1.19–1.22)], followed by two courses [HR 1.13, 95% CI (1.11–1.14)], and a single dose [HR 1.08, 95% CI (1.07–1.09)] [19]. Similarly, the risk of developing AD when antibiotics were administered within the first 90 days of life varies depending on the antibiotics: macrolides were associated with the highest risk of developing AD [HR 1.77, 95% CI: 1.69–1.86], followed by cephalosporin [HR 1.70, 95% CI (1.56–1.85)], penicillin [HR 1.70, 95% CI (1.67–1.73)], and sulfa drugs [HR 1.46, 95% CI (1.37–1.56)] [4]. Interestingly, the risk of developing AD was higher in children whose mothers did not have a history of AD when compared to children with maternal AD [4]. Collectively, these studies suggest that antibiotics in both *in utero* and in early life could influence the development of infantile AD.

## Current Treatments

Treatment of infantile AD depends on both the severity and location of the affected areas. For all patients, regardless of severity, frequent skin hydration, use of thick fragrance-free emollients, and avoidance of potential skin triggers are recommended to maintain the skin barrier [48–52]. For mild cases that are uncontrolled with emollients alone, the 2023 American AAAAI/ACAAI JTF Atopic Dermatitis guidelines suggest applying low-potency topical corticosteroids (TCS), topical calcineurin inhibitors (TCI), or the PDE-4 inhibitor crisaborole for patients 3 months of age and older. Adverse effects of crisaborole include application-site discomfort and possible exacerbation of AD. Calcineurin inhibitors carry a black box warning for an increased risk of lymphoma [53]. Examples of low-potency TCS include alclometasone dipropionate 0.05% ointment and fluocinolone acetonide 0.01% cream [52]. TCS can cause atrophic changes, secondary bacterial infections, hyperpigmentation, and topical steroid withdrawal (TSW), with higher risk associated with higher potency agents [54, 55].

For moderate disease or for patients unresponsive to initial therapy, higher potency corticosteroids are recommended for body areas other than face, neck, and skin folds, which are more susceptible to atrophy and should be treated only with low- or medium-potency steroids. TCIs and PDE-4 inhibitors are safe to use on the face and in skin folds because they are non-steroidal [50, 52, 54, 56]. Examples of higher potency TCS include betamethasone dipropionate 0.05% ointment and fluticasone propionate 0.005% ointment. Short-term use of bandages and emollients may also be considered [57]. Bleach baths may also be considered in moderate cases of AD. If improvement is seen, maintenance includes twice-daily emollient application with twice-weekly TCS use or a TCI.

If there is still no improvement with higher potency corticosteroids, biologics such as dupilumab, a monoclonal antibody targeting IL-4 and IL-13, can be initiated in patients 6 months and older. Potential adverse effects include facial and neck erythema, conjunctivitis, dry eyes, alopecia, and arthritis [58]. Systemic corticosteroid therapy may be considered.

Although not yet formally recognized by the 2023 AAAAI/ACAAI JTF Atopic Dermatitis guidelines, other PDE-4 inhibitors, such as roflumilast, have been proven to be safe and effective in children aged 2–5 years and are currently in clinical trials for children 3 months to 2 years old [59, 60]. Clinical trials have shown that roflumilast helps reduce early pruritus in patients aged 2–5 years and is associated with application-site pain and nasopharyngitis [53]. Another non-steroidal class of therapy under investigation is topical aryl hydrocarbon receptor agonists, such as tapinarof, for children as young as 2 years old. Tapinarof has no restrictions regarding application site or total amount that can be applied, and there is an ongoing trial evaluating its safety and efficacy in patients 3 months to 24 months old [61]. Some associated side effects include mild folliculitis, headache, and nasopharyngitis [62].

Another clinical trial is evaluating the safety and effectiveness of a biologic agent targeting patients 6 months and older with moderate-to-severe AD [63]. This monoclonal antibody binds to IL-13, an interleukin integral to the AD response, and has been shown to reduce systemic inflammatory markers [64]. Reported side effects include conjunctivitis, nasopharyngitis, and headache [65].

Furthermore, Traditional Chinese Medicine (TCM) has demonstrated a promising role in the treatment of pediatric AD [66–68]. TCM consists of herbal formulations administered orally, topically, or as bath therapy. Our group has shown that TCM treatment reduced sleep disturbances, antihistamine use, antibiotic use, and both topical and oral steroid use in patients with severe AD. Specifically, in patients with moderate-to-severe AD treated with triple TCM therapy, we observed reductions in SCORAD scores and topical steroid use after one month of therapy. Given that monoclonal antibodies are costly, carry a risk of relapse upon discontinuation, and are not approved for patients under six months, TCM represents a viable alternative for those suffering from IAD. Since TCM has demonstrated a favorable safety and efficacy profile, we propose its use in IAD even before prior to the introduction of topical steroids.

### Summary

**Fig. 2:**
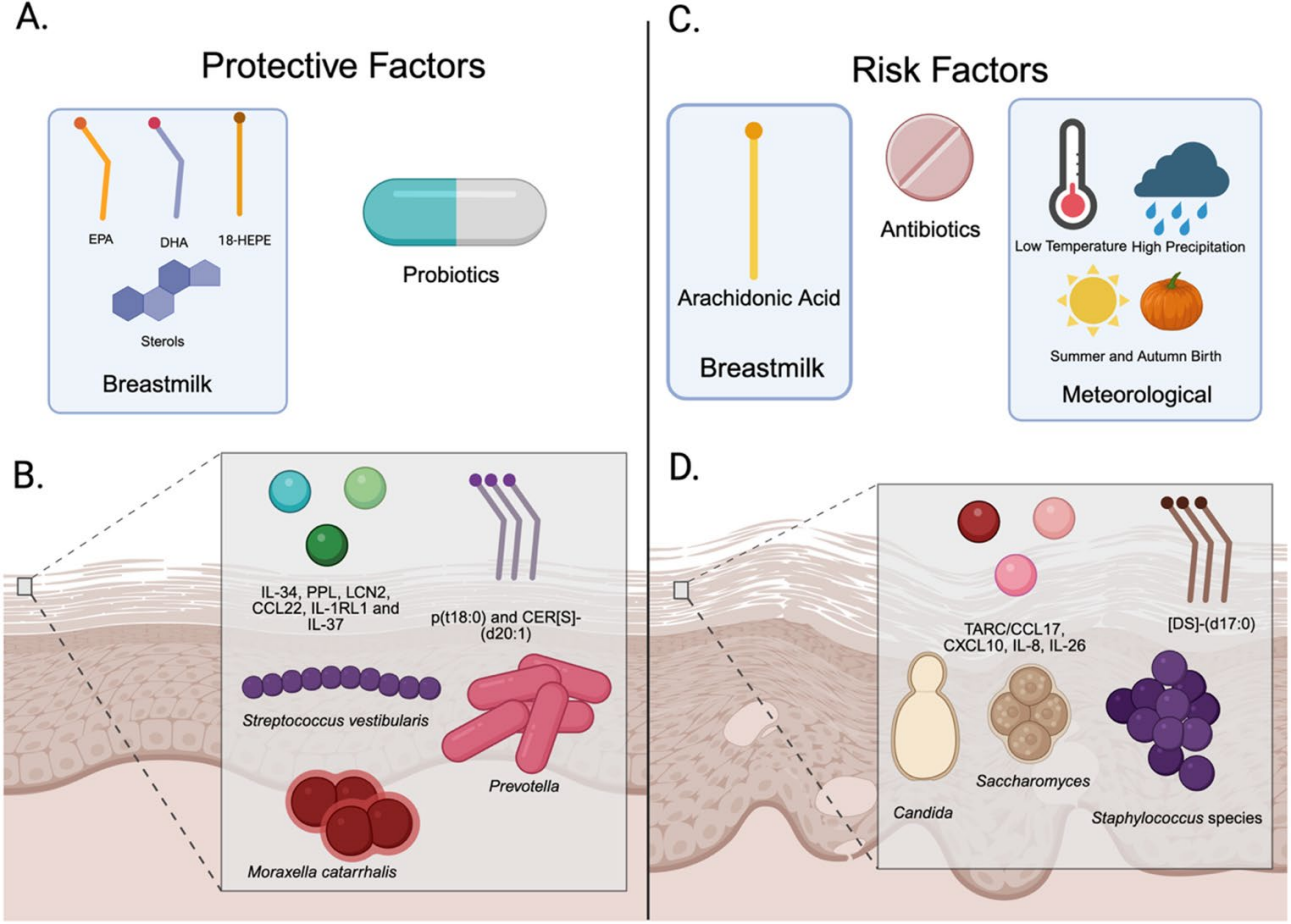
Summary of factors that impact IAD. Protective factors, on the left, and risk factors, on the right, for infantile AD. (**A**) External protective factors include the lipids EPA, DHA, and 18-HEPE in breast milk that are protective. Probiotics are also identified as a protective factor. (**B**) Some intrinsic protective factors include cytokines IL-34, PPL, LCN2, CCL22, IL-1RL1, and IL-37, as well as lipids p(t18:0) and CER[S]-(d20:1). The bacteria *Streptococcus vestibularis*,* Prevotella*, and *Moraxella catarrhalis* also serve as protective factors. (**C**) External risk factors include arachidonic acid is a risk factor, low temperatures, high precipitation, and summer and autumn births, which are associated with the development of IAD. Antibiotics are another risk factor. (**D**) Some intrinsic risk factors include cytokines TARC/CCL17, CXCL10, IL-8, IL-26, and the lipid [DS]-(d17:0). Microbial factors such as *Candida*,* Saccharomyces*, and various *Staphylococcus* species are also associated with increased risk. (Created with BioRender)

Infantile AD can be a troublesome skin condition that can cause extreme distress for both the patient and their parents. Some risk factors are known, such as having a relative with atopy, but there is still more exploration needed in terms of the genetic role in atopy. A summary of these risk factors is in Table 1; Fig. 2.

**Table 1:** Summary of Factors Influencing AD in Infants

Factors	Major Findings	References
Gut-Microbiome	• No maternal intervention with probiotics associated with increased risk of infantile AD. • Higher levels of *Clostridia* found in the IAD group. • Maternal administration of probiotics may be a protective factor in IAD.	Puisto et al. (2025) [12]
	• *Verrucomicrobia* was present only in the maternal and infant healthy groups.	Sung et al. (2022) [13]
	• Infants with colic are at higher risk of developing AD.	Stokholm et al. (2024) [24]
Maternal and Infant Diet	• The concentration of AA in breastmilk is higher in the AD group. • Levels of EPA, 18-HEPE, and DHA in breastmilk are lower in the AD group.	Jiang et al. (2024) [9]
	• Sterols in breastmilk play a protective role in AD.	Van Brakel et al. (2022) [28]
	• Alpha and beta diversity of breastmilk differed between the AD and non-AD groups	Swaney et al. (2024) [14]
	• TAAF and AAF had reduced SCORAD scores	Li et al. (2025) [29]
Meteorological Variable	• Infants born in summer and autumn have higher risk of developing IAD.	Tsuchida et al. (2023) [5]
	• Studies have conflicting evidence on whether sunshine duration and humidity plays a role in AD. • Short duration of sunshine and low humidity combined had a higher risk of developing AD.	Yokomichi et al. (2021) [6]
	• Low maximum and minimum temperatures increase the risk of AD.	Yokomichi et al. (2022) [35]
	• Atmospheric pressure was associated with increasing risk of developing IE. • Higher precipitation decreases the risk of developing IE.	Belugina et al. (2024) [15]
Biomarkers	• TARC/CCL17 and [DS]-(d17:0), were elevated in infants that develop AD. • P(t18:0) and CER[S]-(d20:1) were found to be lower in infants that develop AD	Rinnov et al. (2023) [16]
	• Elevation of TARC/CCL17, CXCL10, IL-8, IL-17a, CCL2, TNFb, IL-18, IL-22, and VEGF in the skin of infants who eventually developed AD • IL-34, PPL, LCN2, CCL22, IL-1RL1 and IL-37 were found to be lower in infants that develop AD	Renert-Yuval et al. (2021) [17]
	• There is a reduction of skin microbial diversity in infants with AD	Halling et al. (2023) [39]
	• Skin barrier dysfunction and reduced filaggrin in skin contributes to overgrowth of *S. aureus*	Paller et al. (2024) [44]
	• Reduction of microbial such as *Prevotella* and elevation of *Fusobacteria*, *Candida*, and *Saccharomyces* in the skin of infants who eventually developed AD.	Chaudhary et al. (2023) [10]
	• Elevation of *Staphylococcus epidermidis* and *Staphylococcus hominis*, while reduction of *Streptococcus vestibularis and Moraxella catarrhalis* were observed in infants prior to clinical onset of AD	Fonfara et al. (2024) [11]
Antibiotics	• Positive association between GBS-IAP administration and AD	Hong et al. (2022) [18]
	• Positive association between maternal antibiotic use during pregnancy and increased risk of AD in infants, with a higher risk in the first trimester.	Tai et al. (2024) [19]
	• Exposure to antibiotics during the first 90 days of life increases the risk of AD with macrolides, with the highest, and Sulfa drugs the lowest.	Fuxench et al. (2024) [4]

More recent studies have examined whether there is maternal or early-life exposure to certain factors that could be associated with the development of AD. Maternal intake of probiotics may help decrease the development of IAD, even though there were no major differences in the gut flora between the IAD and healthy groups. In addition, *Clostridia* were found in higher abundance in the gut of infants with AD [12]. Perhaps this association between the elevated *Clostridia* and IAD could be related to a previously established relationship between the reduced ratio of *bifidobacteria* to *Clostridia* and the risk of developing atopy [12]. A*kkermansia muciniphila* is another bacterium of notable significance due to its presence in higher abundance in the guts of both mothers and infants without AD [2].

Similarly, there have been differences between the breastmilk of mothers with infants with AD and without AD. One notable difference is that mothers with infants were found to have higher concentrations of AA. At the same time, sterols, EPA, 18-HEPE, and DHA had reduced levels in the breastmilk of the AD group, indicating they have anti-inflammatory effects [9, 28]. Another difference is variability in diversity between the breastmilk of mothers of AD infants and non-AD infants [9]. Interestingly, increased formula feeding and a reduction of breastfeeding reduce the risk of AD development. When examining different formulas, the TAAF and AAF formulations also reduce the risk of IAD [29].

When considering different meteorological variables and their influence on IAD, summer and autumn have a higher risk of developing IAD [5]. While there is still conflicting evidence on whether sunshine duration and humidity play a role in AD [6]. However, when taking into consideration the combination of a short duration of sunshine and low humidity, the combination had a higher risk of developing AD [6]. On the other hand, low maximum and minimum temperatures and high atmospheric pressure increase the risk of AD [35]. While higher precipitation decreases the risk of developing IE [15].

Skin biomarkers can be helpful, as there was a difference in pre-clinical IAD and non-IAD skin. There was elevation of TARC/CCL17, CXCL10, IL-8, IL-17a, CCL2, TNFb, IL-18, IL-22, and VEGF and a reduction of IL-34, PPL, LCN2, CCL22, IL-1RL1, and IL-37 in infants who develop AD [16, 17]. Additionally, there was an elevation of *Fusobacteria*, *Candida*,* Saccharomyces*,* S. aureus*,* Staphylococcus hominis*, and *Staphylococcus epidermidis*, while a reduction of *Prevotella*,* Streptococcus vestibularis*,* and Moraxella catarrhalis* was observed in infants before the clinical onset of AD [10, 11].

Antibiotics may also influence the risk of IAD both perinatally and in early-life exposure. There is a positive association between maternal antibiotic use during pregnancy and increased risk of AD in infants, with the highest risk in the first trimester [18, 19]. Additionally, exposure to antibiotics during the first 90 days of life increases the risk of IAD, with macrolides associated with the highest risk [4].

## Conclusion

This mini-review focuses on the risk and protective factors of IAD. Past research examined risk factors generally for AD but did not specifically address infantile cases or variables like breastmilk and meteorological factors [69]. Current literature suggests the gut microbiome is important in IAD development, but more research is needed to understand how breastmilk influences the gut and skin microbiomes. Some studies suggest that components like arachidonic acid may increase the risk of IAD, possibly through inflammatory products derived from metabolism or gut bacteria. Environmental factors could guide physician recommendations for non-pharmacological interventions like emollients to their patients to help protect the skin barrier and prevent and reduce the symptoms of IAD. Identifying the various pre-clinical cytokines and biomarkers can help identify infants at risk for IAD. Further research is warranted into ways to increase beneficial skin bacteria as a form of prevention. Awareness of perinatal and early-life antibiotic exposure also opens avenues to predict and possibly prevent AD through microbiome analysis. In conclusion, while many studies examine risk factors for AD generally, more work is needed to understand how early-life exposures interplay and which interventions could best reduce the risk of IAD.

## Key References

- Fuxench ZC, Mitra N, Del Pozo D, Hoffstad O, Shin DB, Langan SM, et al. In utero or early-in-life exposure to antibiotics and the risk of childhood AD, a population-based cohort study. Br J Dermatol. 2024;191(1):58–64. doi: 10.1093/bjd/ljad428.
  - ◦A cohort study involving a large patient population, including infants with AD and prenatal maternal antibiotic exposure. The study considers several factors, such as the types of antibiotics used and the trimester when they were administered.
- Chaudhary PP, Myles IA, Zeldin J, Dabdoub S, Deopujari V, Baveja R, et al. Shotgun metagenomic sequencing on skin microbiome indicates dysbiosis exists prior to the onset of AD. Allergy. 2023;78(10):2724–31. doi: 10.1111/all.15806.
  - ◦ A cohort study that collected skin swabs from infants during their first week of life and then followed them for three years to determine the development of AD. This study examines how the variations in the skin microbiome might influence the outcomes of AD.

## Data Availability

No datasets were generated or analysed during the current study.
